# Left-Sided Laparoscopic Appendectomy in a Pediatric Patient With Situs Inversus Totalis

**DOI:** 10.7759/cureus.35844

**Published:** 2023-03-06

**Authors:** Kiley A Fincher, Mohamed R Bakeer

**Affiliations:** 1 General Surgery, Edward Via College of Osteopathic Medicine-Louisiana Campus, Monroe, USA; 2 General Surgery, St. Francis P & S Surgery & Heart Center, Monroe, USA

**Keywords:** pediatric surgery, laparoscopic appendectomy, kartagener's syndrome, situs inversus, left-sided acute appendicitis

## Abstract

Acute appendicitis classically presents as periumbilical pain that migrates to the right lower quadrant. Rarely, left-sided appendicitis can occur, but it is not commonly considered in the differential of left lower quadrant pain. This report intends to raise awareness of left-sided appendicitis, in this case, due to situs inversus totalis, and to emphasize the need to perform a thorough patient evaluation. Here, we discuss the case of a 10-year-old male with known situs inversus totalis and primary ciliary dyskinesia (suspected Kartagener’s syndrome) who presented to the emergency room with a one-day history of left lower quadrant pain and associated nausea and vomiting. His white blood cell (WBC) count was elevated, and a contrast-enhanced computed tomography (CT) scan revealed acute tip appendicitis in the left lower quadrant. The surgeon performed a laparoscopic appendectomy with modifications. The patient tolerated the procedure well but experienced difficulty weaning off oxygen postoperatively. An airway management plan was initiated, which allowed for the discontinuation of oxygen. The patient was discharged on postoperative day two and was seen in the clinic approximately two weeks later with no postoperative complications. Pathology confirmed acute suppurative appendicitis.

## Introduction

Appendicitis is a common cause of an acute abdomen and classically presents as right lower quadrant pain following periumbilical discomfort [1]. Left-sided appendicitis is rare and can be overshadowed by the wide differential that usually accompanies left lower quadrant pain. In pediatrics, this includes but is not limited to mesenteric adenitis, ovarian/testicular torsion, Meckel’s diverticulum, gastroenteritis, incarcerated hernias, constipation, and lactose intolerance [2]. A complete medical history, physical exam, laboratory tests, and imaging are paramount to reaching the correct diagnosis in a timely manner. A delay can lead to the development of complications such as abscesses, perforations, and peritonitis [3]. Three congenital conditions that can predispose a patient to develop left-sided appendicitis are situs inversus, malrotation of the gut, and a majorly elongated appendix [1,4-5]. Situs inversus, though, is responsible for greater than 67% of left-sided appendicitis cases [4-5]. 

Nonetheless, a PubMed search using the phrase “pediatric left-sided appendicitis” revealed few pediatric case reports, and of the pediatric case reports reviewed, most detailed congenital malrotation of the gut as the etiology for left-sided appendicitis. Here, we discuss left-sided appendicitis due to situs inversus totalis in a pediatric patient.

## Case presentation

*Written informed consent was acquired from the patient’s mother for this case study.

A 10-year-old male with known situs inversus totalis and primary ciliary dyskinesia (suspected Kartagener’s syndrome) presented to the emergency department with a one-day history of left lower quadrant pain with associated nausea and vomiting. The patient reported having regular bowel movements. His vitals upon presentation are shown in Table 1.

**Table 1:** Patient’s Vitals Upon Presentation (08/03/2022)

Blood Pressure	Pulse	Temperature	Respiratory Rate	Height	Weight	Oxygen Saturation	Body Mass Index
101/52	110 bpm	97.8 °F	18 bpm	57 in	103 lbs	99%	22.29 kg/​​m²

Physical examination revealed a soft, non-distended abdomen with tenderness to palpation over the left lower quadrant. Top differentials considered for this patient included acute appendicitis, Meckel's diverticulum, mesenteric adenitis, gastroenteritis, pancreatitis, and urinary tract infection. Lipase was within normal limits. Urinalysis was negative for leukocyte esterase and nitrites. Abnormal complete blood cell count (CBC) results were as follows in Table 2.

**Table 2:** Patient’s Abnormal CBC Lab Results (08/03/2022)

Component	Reference Range & Units	Patient Lab Value
White Blood Cell Count	4.3-11.0 1000/uL	20.1 1000/uL High
Hematocrit	32.2-39.8%	40.2% High
Neutrophils Abs	1.6-7.6 1000/uL	17.6 1000/uL High
Monocytes Abs	0.2-0.9 1000/uL	1.1 1000/uL High
Neutrophils %	29%-75%	88% High
Lymphocytes %	16%-57%	7% Low
Immature Grans (Abs)	0.00-0.04 1000/uL	0.07 1000/uL High

**Figure 1:**
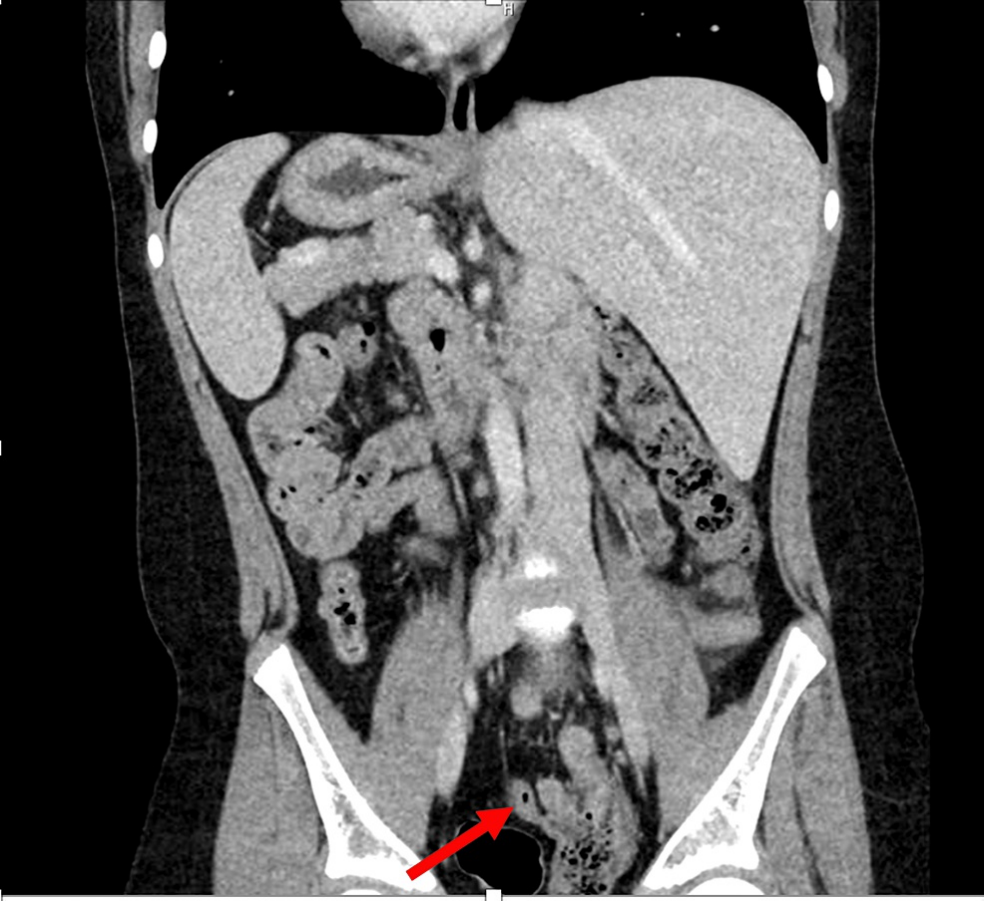
CT scan (coronal view). The red arrow points to the thickening of the appendix located on the patient's left side, suggestive of acute tip appendicitis. Upon excision, the specimen measured 7.5 x 1.2 cm.

**Figure 2:**
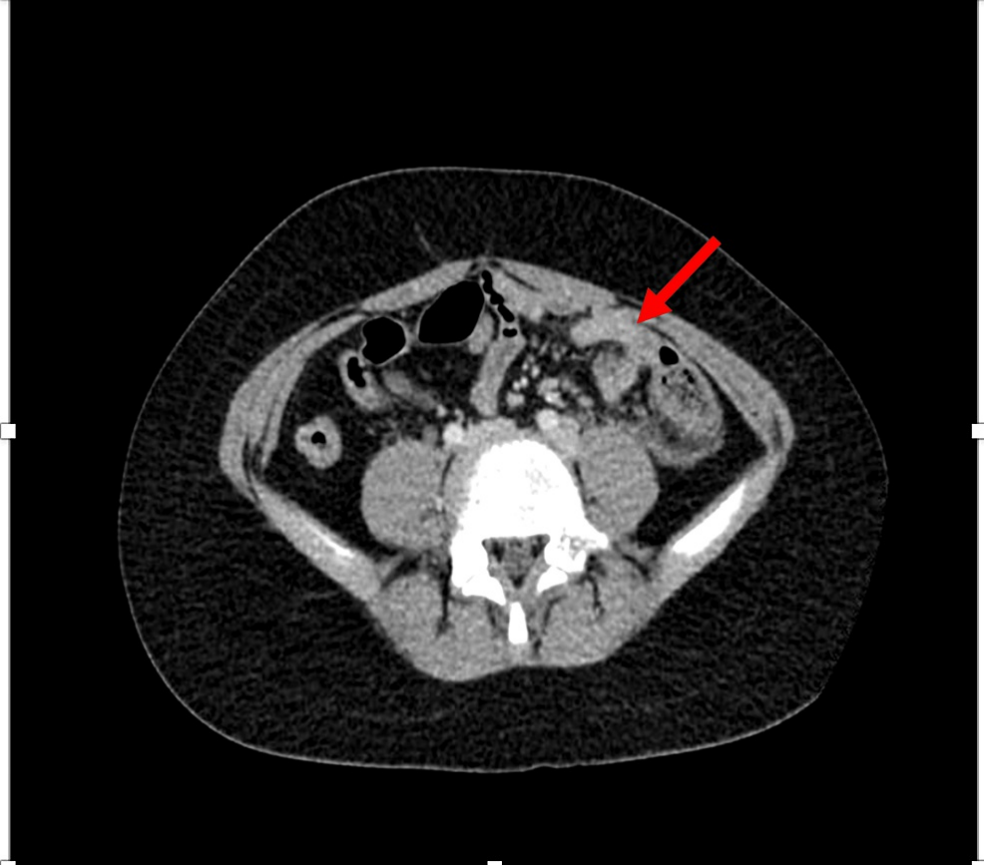
CT scan (axial view). The red arrow points to the thickening of the appendix located on the patient's left side, suggestive of acute tip appendicitis. Upon excision, the specimen measured 7.5 x 1.2 cm.

Computed tomography (CT) imaging of the abdomen and pelvis was ordered in the emergency room. The radiology report revealed acute tip appendicitis with mild reactive free fluid in the left lower quadrant and pelvis. No abscess seen or pneumoperitoneum. The CT views are shown in Figures 1, 2.

The risks, benefits, indications, and alternatives to a laparoscopic appendectomy were discussed with the patient's parents. After gaining consent to the procedure, the patient was taken to the operating room. The patient was placed supine, and the abdomen was both prepped and draped using a sterile technique. The surgeon stood on the patient’s right side. To begin, a 5 mm trocar was placed above the umbilicus using the Veress technique. The abdomen was then insufflated to 12 mmHg, and the camera was placed in the abdomen with no injuries upon entry. Another 5 mm trocar was placed midline suprapubically, and a 12 mm trocar was placed in the left lower quadrant. The appendix was visualized and pulled toward the anterior abdominal wall. The appendix appeared both inflamed and injected. The cecal base, however, was normal, and no pus was seen in the peritoneal cavity. Next, a window was created between the appendix and mesoappendix. The appendix and mesoappendix were resected using stapler loads. The specimen was sent to pathology. Hemostasis was ensured, and the free fluid was suctioned. The fascia was closed using a 0 Vicryl, and the skin sites were closed using 4-0 Monocryl. The procedure took 1 hour and 19 minutes. (This paragraph was extracted from the surgeon’s intraoperative notes).

The patient tolerated the surgery well and was transferred to the post-anesthesia care unit in stable condition. However, the patient required oxygen postoperatively. While these oxygenation issues may have been due to temporarily impaired pulmonary function as a result of anesthesia and mechanical ventilation, the patient’s underlying respiratory problems from primary ciliary dyskinesia (suspected Kartagener's syndrome) predisposed him to develop hypoxia after surgery. To assist the patient, the pediatric hospitalist worked in tandem with the patient’s pulmonologist to implement an airway clearance plan. The airway clearance plan involved albuterol every four hours followed by a 3% saline nebulizer every eight hours, Flovent, Aerobika device every 3 to 4 hours as needed, huff cough, chest physiotherapy, and ambulation, which eventually resulted in the discontinuation of oxygen before leaving the hospital. The patient was discharged on postoperative day two afebrile; his pain was well controlled, and he tolerated his diet without nausea or vomiting. He was instructed to follow up with his surgeon in the clinic two weeks later. 

At his follow-up, the patient had no postoperative complaints. Physical examination revealed that the abdomen was soft, non-tender, and non-distended, and the incisions were dry, clean, and intact with no erythema or drainage. Pathology confirmed acute suppurative appendicitis. 

## Discussion

The patient’s medical history revealed situs inversus totalis and primary ciliary dyskinesia (suspected Kartagener’s syndrome). Primary ciliary dyskinesia is an autosomal recessive disorder that affects ciliary motility characterized by recurrent respiratory infections and fertility problems [6]. In 50% of cases, primary ciliary dyskinesia occurs in combination with situs inversus and is usually referred to as Kartagener’s syndrome [6]. Kartagener’s syndrome occurs in one in 30,000 people and involves a mutation in two genes, DNAI1 and DNAH5, which “cause the cilia to be the wrong size or shape or move in the wrong way, making ciliary motility defective” [7-11]. The proper ciliary function is necessary for determining the laterality of organs during embryonic life [9]. When ciliary motility is impaired, situs inversus occurs, and the appendix settles on the left side.

The incidence of left-sided appendicitis in a patient with situs inversus is “exceedingly low” at 0.016% to 0.024% [12]. To make the diagnosis of left-sided appendicitis, physicians must have a high clinical index of suspicion. A complete history, physical examination, bloodwork, and imaging are crucial to diagnosis. Ultrasound is often chosen in pediatrics to limit radiation exposure. CT imaging, however, is more precise and has a 90%-98% accuracy rate in acute appendicitis cases [12]. CT findings of uncomplicated appendicitis are the same regardless of laterality: a dilated appendix with a diameter larger than 6 mm, appendiceal wall thickening, and peri appendiceal inflammation [13].

Without treatment, the appendix can burst, and infection can spread. Two ways to treat appendicitis include antibiotics and surgery. The durability of nonoperative management of appendicitis in pediatric patients over time is not yet fully understood. Studies show that “[a]mong U.S. pediatric hospitals, one-third of these children are medically managed and experience higher rates of subsequent healthcare utilization and perforation” [14]. Surgery is the definitive treatment for appendicitis. It removes the appendix, and no further appendicitis will occur. However, surgery also has associated risks. The risks and benefits of each approach should be independently weighed and considered. 

In this instance, laparoscopic surgery was performed. In the case of laparoscopic surgery with left-sided appendicitis, important adjustments must be made in the operating room. The surgeon stands on the right side of the operating table instead of the left, and the monitor is placed on the patient’s left side across from the surgeon. The trocars are placed according to the position that allows the best visual access to the left side. Because this is not a routine procedure, “there is no standard position for the insertion of trocars in these particular cases, and port placement should be modified by the surgeon following laparoscopic surgery principles” [12,15-16]. Postoperative care remains the same as a routine appendectomy in these patients. 

## Conclusions

In conclusion, left-sided appendicitis in pediatric patients is rare and can be overshadowed by the wide differential that usually accompanies left lower quadrant pain. To make the correct diagnosis, physicians must be aware of this anatomical anomaly as well as its etiology. In situations where laparoscopic surgery is appropriate, adjusting the surgeon's position, the operating room setup, and the surgical devices is vital to a successful outcome. 
